# 

**Published:** 1955-11

**Authors:** 


### Contents

| | | Page |
|---|---|---|
| THAT BACKBONE . . . . | *Dr. A. Gordon Heron* | 183 |
| HOME CARE OF THE DIABETIC . . | *Dr. C. T. Andrews* | 193 |
| CHILD LIFE IN PAST TIMES . . . | *Dr. S. W. Hinds* | 199 |
| THE CHANGING PATTERN OF PULMONARY TUBERCULOSIS . | *Dr. R. A. Craig* | 206 |
| THE GENERAL AND THE SPECIAL IN MEDICAL PRACTICE | *Dr. E. M. Bluestone* | 215 |
| HAEMORRHAGE FROM A CAVERNOUS HAEMANGIOMA OF THE CORD CLINICALLY RESEMBLING AN ASCENDING MYELITIS | | 219 |
| NEWS FROM SOCIETIES . . . . . . . | | 222 |
| ANNOTATIONS . . . . . . . . . | | 225 |
| NOTES AND NEWS . . . . . . . | | 226 |
| APPOINTMENTS . . . . . . . . . | | 226 |

